# Reaching the Unreached: Bridging Islam and Science to Treat the Mental Wounds of War

**DOI:** 10.3389/fpsyt.2021.599293

**Published:** 2021-06-02

**Authors:** Lori A. Zoellner, Jacob A. Bentley, Norah C. Feeny, Alexandra B. Klein, Michael L. Dolezal, Dega A. Angula, Momin H. Egeh

**Affiliations:** ^1^Department of Psychology, University of Washington, Seattle, WA, United States; ^2^Department of Clinical Psychology, Seattle Pacific University, Seattle, WA, United States; ^3^Department of Psychological Sciences, Case Western Reserve University, Cleveland, OH, United States; ^4^Ma'alin Haruon Masjid, Hargeisa, Somalia; ^5^Abu-Bakar Al-Siddique Islamic Center, Borama, Somalia

**Keywords:** war, trauma, PTSD, Islam, depression, mosque, community, psychotherapy

## Abstract

Radical new paradigms are needed to equip non-professionals and leverage community faith-based infrastructure to address the individual and communal wounds of war- and conflict-related trauma. Muslims in war-torn regions like Somalia experience high rates of trauma and posttraumatic stress; yet, lack of providers, potential stigma, and lack of integration with one's faith are substantial barriers to care. In this pre-post feasibility clinical trial (NCT03761732), mosque leaders implemented a brief, group- and mosque-based intervention, *Islamic Trauma Healing*, targeting trauma-related psychopathology and community reconciliation for trauma survivors (*N* = 26) in Somaliland, Somalia. Leaders were trained in a brief 2-day training, with supervision provided remotely via WhatsApp. This six-session intervention combines empirically-supported trauma-focused psychotherapy and Islamic principles, focusing on wisdom from the lives of the Prophets and turning to Allah in dua about trauma. There were large, clinically meaningful effects for PTSD (*g* = 1.91), depression (*g* = 2.00), somatic symptoms (*g* = 2.73), and well-being (*g* = 1.77). Qualitative data from group members highlighted how well the program was aligned with their Islamic faith, built community, and need to expand the program. These results highlight the feasibility of this non-expert, easily up-scalable mental health approach in war-torn Muslim regions and refugee communities. This program has the potential to provide a low-cost, self-sustaining, Islam-based intervention addressing the psychological wounds of war consistent with the IOM's call to develop novel approaches to address unmet clinical needs.

**ClinicalTrials.gov** **Identifier:** NCT03761732.

## Introduction

“Without mental health there can be no true physical health.” Dr. Brock Chisholm, the first Director-General of the World Health Organization, famously stated this more than 60 years ago (1). Despite decades of advancement, evidence-based mental health interventions have failed to permeate low- and middle-income countries (LMICs) or refugee populations (2). In Somalia, there is next to no community mental health care (3). Further, there have been no documented in-country mental health clinical trials of any evidence-based interventions.

Trauma-related mental health needs in the Islamic world are staggering. Of the six civil wars in the world, five are in Islamic nations, leaving over 68.5 million displaced peoples (4). Muslims in, and refugees from, war-torn regions like Somalia experience high trauma burden and are at an increased risk for posttraumatic stress disorder (PTSD) and related impairment (5, 6). The international community faces a crisis in addressing mental health needs of those in war-torn LMICs and resultant refugee populations (7).

Effective PTSD interventions exist. Cognitive- and exposure-based psychotherapies have the strongest evidence-base across diverse samples (8), including refugees and those in sub-Saharan Africa e.g., (9). Yet, barriers to receiving state-of-the-art care exist (10). Scalable, cost-effective interventions cannot rely on extensively-trained experts or existing infrastructure (11). Even when PTSD interventions are implemented in LMICs, they rely on time-consuming training models across weeks of on-the-ground presence and months of careful supervision (e.g., 6 weeks lay leader training) (9). Despite ~23% of the world being Muslim (12), no existing PTSD interventions are Islamic focused. Mental health care utilization is low among Muslim communities; partially due to perceived incongruence with Islam (13) and deeply entrenched stigma associated with seeking mental health care. Religious practices are often seen as a first-line intervention, and many Muslims primarily use private prayer to cope following war trauma (14).

The development and implementation of evidence-based paradigms aligned with the Qur'an, communal culture, and broadly applicable for war-torn and refugee Muslim communities is critically important. Further, the advent of smartphone and internet infrastructure in LMICs enables new models of “reach” between intervention experts and communities. Islamic Trauma Healing is a brief, six-session group- and mosque-based, lay-led intervention that targets trauma-related psychopathology and community reconciliation. Through an iterative development process, the following factors were identified as crucial for uptake: centrality of Islam in life, reducing mental illness stigma, promoting reconciliation and social connectedness, and empowering the community to facilitate trauma healing (15). A PTSD-specific, targeted mechanism approach was applied incorporating evidence-based principles of change, namely shifting unhelpful beliefs, reducing trauma-related avoidance, and enhancing social support (16). By focusing on these “treatment drivers,” the intervention aims to be targeted and efficient. A pilot trial in a U.S.-based Somali refugee sample (17) supports the acceptability and preliminary efficacy of this intervention across PTSD, depression, somatic symptoms, and well-being (Hedges' gs = 0.76–3.22).

The lay-led group structure of the program seeks to promote community building, acknowledge trauma's impact in the community, and build infrastructure for wider uptake. The program is not referred to as “therapy” or “treatment” for “mental illness.” Each session includes time for community building rituals (e.g., tea, incense), spiritual preparation using a brief supplication written by the local Imam, prophet narratives and group discussion, individual time spent turning to Allah in dua (i.e., informal prayer) about trauma memories, and a brief closing supplication (18). In each session, narratives of prophets who experienced trauma present Islamic principles, verses from the Qur'an, and facilitate cognitive shifts. Group discussion questions then promote shifting of thinking about trauma (e.g., “*How do you think Yusuf [Joseph] would feel when his brothers betrayed him? Where was Allah in the midst of Yusuf's trials? What did Yusuf do with his betrayal (even when it was done by his family)?”)*, incorporating cognitive restructuring as a therapeutic technique. Group members spend individual time turning to Allah in dua, focused on approaching rather than avoiding trauma memories. This informal prayer incorporates the exposure-based therapeutic technique of imaginal exposure, where the trauma memory is revisited in detail (19). Clear instructions are given for dua at each session and the group reconvenes to discuss how the time turning to Allah went and lessons learned (e.g., “*How does admitting our fears and concerns to Allah help with our healing? How does this relate to Musa [Moses] overcoming his fears?”*). This illustrates a fundamental shift and new model for in-depth integration of Islam and evidence-based PTSD treatment, bridging their collective wisdom to provide trauma-related mental health treatment.

To reduce stigma, Islamic Trauma Healing utilizes local partners embedded within the strong traditional, communal mosque infrastructure. In accordance with cultural norms, men's and women's groups are separate with same-gender leaders. To be sustainable easily scalable, lay leaders do not require extensive expertise or training. The program follows a train-the-trainer model, led by current group leaders, empowering lay leaders to facilitate healing in their communities. Indeed, sustainable interventions must have the capacity to be delivered in the at-risk population utilizing pre-existing resources (20). Indeed, identified community ownership and mobilization have been identified as crucial facilitators underlying health-care sustainability (21). Community-based programs that utilize train-the-trainer methods may be particularly well-suited to address stigma and accessibility challenges.

To test Islamic Trauma Healing's feasibility and preliminary effectiveness, we conducted a pre-post clinical trial in Somalia. We hypothesized that the intervention would reduce PTSD, depressive, and somatic symptoms while enhancing overall well-being.

## Materials and Methods

### Participants

Somali men (*n* = 12) and women (*n* = 14) aged 18–65 participated in the study. Inclusion criteria were exposure to at least one DSM-5 Criterion A trauma (22) and presence of at least one PTSD symptom, specifically either avoidance or re-experiencing. All participants were of the Islamic faith. Participants were excluded if they were visibly cognitively impaired or had current suicidal intent.

Twenty-eight were recruited, signed consents, and participated in the groups. Two men did not report any re-experiencing or avoidance symptoms and thus were excluded from analysis, resulting in a final sample size of *N* = 26. On average, participants were 27.62 years of age (*SD* = 7.35), ranging from 18 to 47 years old. The majority reported being single (69.2%), with 19.2% being married, and 11.5% reporting that there were divorced or separated. Approximately half of the sample had a college education (53.8%). There was heterogeneity in terms of self-identified DSM-5 primary target trauma exposure: military combat or living in a war zone (30.8%); natural disaster (19.2%); physical assault (15.4%); sexual assault in adult or childhood (11.5%); accident (11.5%); or other trauma (11.5%).

### Islamic Trauma Healing Intervention

The program contained six sessions. This duration is in line with evidence that brief protocols can substantially reduce PTSD and other trauma-related symptoms (23). Key components of the program included psychoeducation, community building, prophet narratives (i.e., cognitive restructuring), and turning to Allah in dua (i.e., imaginal exposure), targeting trauma-related beliefs and reducing trauma-related avoidance. A manual for the program was developed (18) with input from local Imams, community members, and a university-based Islamic scholar, with an eye toward avoiding sectarian disputes, focusing on central Islamic principles, and facilitating broader uptake in the Muslim world. Focus group feedback from lay leaders and group members was also utilized in revisions. The program was designed for groups of five to seven members, with two lay leaders of the same gender. The manual contained clear instructions to assist group leaders in progressing through session-by-session content. Each session included time for community building rituals (e.g., sharing tea and snacks), spiritual preparation using a brief supplication written by an Imam, prophet narratives relevant to trauma healing, and a brief closing supplication also written by an Imam. The first session included a rationale for the program, common reactions to trauma, an initial prophet narrative and discussion, and a breathing relaxation exercise. Turning to Allah about the trauma in dua (i.e., informal prayer) was conducted from the second to last session. In the last session, group members were encouraged to organize a closing event, at which certificates of program completion were given.

#### Prophet Narratives and Group Discussion

Prophet narratives were brief, thematically focused synopses that include Qur'an verses. Group questions mirroring the theme for the session were discussed to facilitate cognitive restructuring. Prophet narrative content and questions shifted across sessions from the presence and purpose of suffering to healing and reconciliation for oneself, others, and the larger community. In each session, narratives of prophets who have undergone trauma (e.g., Prophet Ayyub [Job]: faith during hard times; Prophet Musa [Moses]: overcoming fear) presented Islamic principles, verses from the Qur'an, and facilitated cognitive shifts. Below is a partial example of prophet narrative content.

Allah said about Ayyub, “*Truly! We found him patient. How excellent a slave! Verily, he was ever oft returning in repentance to Us!” (Qur'an 38:44)*. Prophet Ayyub was a very rich man with many children. He had a lot of cattle, sheep, and lands of great farms. Suddenly, Ayyub lost all of his children and wealth. Not only that, he was also afflicted with a strange disease with painful sores from head to toe. Only his tongue and heart were saved from this affliction. This lasted for a long time. His people and relatives deserted him…

Questions for group discussion then promote shifting of thinking about trauma *(e.g., “What was Ayyub's life like during and after his sickness? What is the role of affliction and suffering in faith?”)*, incorporating elements of cognitive restructuring as a therapeutic technique.

#### Turning to Allah in Dua and Group Discussion

From sessions two through six, participants were asked to spend time in individual dua turning to Allah about their trauma. Of note, the term “prayer” is used as an illustration, having varied meanings within Islamic practice, with the manual using the term “turning to Allah in dua.” In the pilot trial (17), the concept of turning to Allah about personal experiences, including trauma, was very intuitive to group leaders and group members alike. In the first session, group leaders provided a rationale for turning to Allah in dua. In the second session, this rationale was repeated, including instructions about how to select a trauma memory, and an example dua was provided. Turning to Allah was conducted individually for ~15–20 min across sessions.

This individual time turning to Allah in dua focused on approaching rather than avoiding trauma memories. This informal prayer incorporated the exposure-based therapeutic technique of imaginal exposure, where the trauma memory is revisited in detail, with content shifting from recounting the most severe traumatic event, to focusing on personal thoughts and feelings, to the hardest parts of the memory, to ultimately thanking and praising Allah for the lessons learned because of the trauma in the later sessions. Below is a partial example of the rationale for turning to Allah in dua about the trauma.

Allah is all knowing. He already knows what has happened to you, knows your suffering, and knows your heart. Nothing you can say to Him will be shocking or surprising.

Yet sometimes we try to hide these things from Him or even from ourselves. We stuff painful memories away and don't let ourselves think about them or feel them. If we think of these memories as being “off-limits” or too difficult or hard to think about, we cannot fully experience the healing Allah intends for us.

Clear “how to” instructions were given for the dua at each session, and individuals reconvened as a group to discuss how the time turning to Allah went and lessons learned (e.g., “*Why might some people be afraid to turn to Allah about what happened to them? What might be some things they could learn about themselves or about Allah while spending time with Him?”*). Group members were encouraged to talk in the group about their experience of the revisiting of the trauma memory but not to directly share the content of the traumatic experiences with the group, with the intention to promote cognitive restructuring of negative trauma-related beliefs and foster social connectedness among group members.

### Leader Training and Supervision

Lay leader training focused on teaching leaders discussion leading skills, with the manual content providing more direct therapeutic work. That is, the leaders were not explicitly taught how to be psychotherapists or do cognitive behavioral therapy specifically. The manual contained an introduction to trauma healing, including a description of types of trauma exposure and common reactions, as well as Islamic principles related to trauma healing.

Lay leaders attended a 2-day training session in Djibouti, Djibouti (~8 h of actual training, allowing for breaks and prayer times), where they received the manual and an overview of the program, reviewed and practiced components of group sessions, and identified and problem-solved potential issues. This in-person training was led by the clinical team (L.A.Z., J.A.B., N.C.F., A.B.K., M.L.D.). Group leaders also received standardized training on confidentiality and common ethical considerations in facilitating a group. The training was provided in English by the research team and translated to Somali by leaders fluent in both English and Somali.

During provision of the groups, lay leader supervision was conducted weekly via WhatsApp, a free cross-platform instant messaging system. After each group, group leaders met with the investigational team via WhatsApp for a discussion of the session, with simultaneous translation for leaders whose English was more limited. In advance, key issues were identified for each session and framed as questions (e.g., “*In this session, participants turned to Allah in dua about the trauma for the first time. How did it go?”*) which structured the supervision and allowed for feedback and trouble shooting.

### Measures

Questionnaires were translated and back-translated from English to Somali, with audio versions in Somali also provided. Questionnaires were completed using a mobile device (e.g., tablet) and headphones. Mental health functioning following trauma assessed in this study was dimensional and multifaceted, examining not only PTSD, but also somatic symptoms, depression, and functional well-being.

The primary outcome measure was self-reported PTSD severity as measured by the PTSD Scale Self-Report for DSM-5 (PDS-5) (24). Additional self-report measures included the Patient Health Questionnaire-9 (PHQ-9) (25), Somatic Symptom Scale-8 (SSS-8) (26), WHO-5 Well-being Index (WHO-5) (27), and the Client Services Satisfaction Questionnaire (28).

### Focus Group Analysis

Semi-structured interviews were conducted in Somali by lay leaders following the final session. Participants were asked five questions: (1) *What do you think about the Islamic Trauma Healing program?*; (2) *What did you most like about the program?*; (3) *Where does the program need improvement?*; (4) *What would keep men or women from being involved in the program? What are barriers to participating?*; and (5) *What lessons have you learned from the program?* All interviews were audio recorded and translated to English by a research team member in Somaliland. Two independent raters, not involved in study procedures or with the qualitative analyses conducted as part of the pilot trial in the United States (17), coded participants' responses. For any discrepancies, the raters came to consensus. Qualitative analysis was conducted using NVivo 12 Pro (QSR International).

### Lay Leaders

Male and female volunteer group leaders were selected by the last author based on being a community leader, someone respected or in leadership within their Islamic center, with a “heart for healing and reconciliation” (e.g., a person who sees the effects of trauma exposure in his or her community and wants to promote healing and reconciliation) in the Somali community, and willingness to later train additional group leaders. Once trained, the same leaders ran the groups from the first to last session. All lay leaders signed contracts to be individual investigators affiliated with the respective university, and all procedures were approved by the respective Institutional Review Board. Permission for the study was obtained from the Somaliland Ministry of National Planning and National Development and the Somaliland Ministry of Endowment and Religion Affairs.

Groups were at two mosques in Borama and Hargeisa, Somaliland, Somalia. Four groups (*n* = 7, each) were conducted in total, with one men's and one women's group run in each city led by two group leaders of matching gender. Participants completed and signed informed consent forms in Somali. Questionnaires were completed at the beginning and end of the six sessions. Groups met weekly for 2 h, with length varying depending on breaks for the call to prayer. Data collection occurred from October 15, 2018, to February 6, 2019.

## Results

### Pre- to Post-Intervention Outcomes

Means, standard deviations, and effects sizes for the main outcome measures using Hedges' g, corrected for use with small samples, are presented in Table 1. As can be seen, there were large intervention effects for PTSD severity (*t*(25) = 9.30, *p* < 0.001), depression (*t*(25) = 9.15, *p* < 0.001), somatic symptoms (*t*(25) = 12.10, *p* < 0.001), and well-being (*t*(25) = −7.11, *p* < 0.001). There was no participant dropout or adverse events. At pre-, 80.8% met symptom criteria for a DSM-5 PTSD diagnosis, and at post-intervention 15.4% met criteria.

**Table 1:** Pre and post group measures of psychopathology and functioning.

**Measures**	**Pre-group (*N* = 26)**	**Post-group (*N* = 26)**	**Hedges' unbiased g**
PTSD severity (PDS-5)	30.88 (13.76)	8.08 (5.74)	1.91
Reexperiencing	8.35 (4.08)	2.31 (1.87)	1.54
Avoidance	3.08 (1.79)	1.11 (0.86)	1.31
Negative thoughts and mood	10.62 (5.72)	2.38 (2.28)	1.61
Hyperarousal and reactivity	8.85 (4.20)	2.27 (1.76)	1.94
Depression symptoms (PHQ-9)	10.42 (4.69)	2.50 (1.50)	2.00
Somatic symptoms (SSS-8)	8.46 (2.53)	2.38 (1.58)	2.74
General well-being (WHO-5)	10.00 (6.62)	19.38 (2.48)	1.77

*PDS-5, Posttraumatic Diagnostic Scale for DSM-5; PHQ, Patient Health Questionnaire; SSS, Somatic Symptoms Scale; WHO-5, Well-being Index*.

Notably, higher pre-treatment severity was associated with larger change from pre- to post-intervention across outcomes (PDS-5: *r* = 0.91, *p* < 0.001; PHQ-9: *r* = 0.95, *p* < 0.001; SSS-8: *r* = 0.81, *p* < 0.001; WHO-5: *r* = 0.93, *p* < 0.001), arguing that the program conferred the strongest benefit for those with more severe symptoms; particularly important for a brief, lay-led intervention. *Post-hoc* analyses showing lack of age and gender effects on post-intervention PTSD severity are available in Supplementary Materials.

### Program Satisfaction

Overall, participants reported being highly satisfied with the program (*M* = 3.88, *SD* = 0.11, range: 3.6–4.0, CSS). Regarding specific questions, responses again were in the excellent range: “*How well do you think the Islamic Trauma Healing program has helped with your trauma-related healing*?” (*M* = 3.96, *SD* = 0.20, range: 3–4); “*How well do you think the Islamic Trauma Healing program will help with community reconciliation*?” (*M* = 3.73, *SD* = 0.45, range: 3–4); “*To what extent did the Islamic Trauma Healing program match with your religious beliefs and cultural practices?*” (*M* = 3.81, *SD* = 0.40, range: 3–4); “*If a friend wanted trauma-related healing and reconciliation, would you recommend this program to him/her?*” (*M* = 4.00, *SD* = 0.00, range: 4–4)*;* and “*Overall, how satisfied are you with the Islamic Trauma Healing program?*” (*M* = 3.88, *SD* = 0.33, range: 3–4).

### Qualitative Impressions of the Program

Three overarching themes emerged from group member feedback, with responses that were overwhelmingly positive and highlighted the need for wider dissemination. A central theme was the Importance of Islam, with participants stating, “What I most liked about this program was the stories of the Prophets” and expressing appreciation for “making healing from the Islamic religion.” One member said, “I knew that dua and [the] Qur'an heal[ed] the invisible damages of the heart and mind [I learned] to do permanently the Dua and turning to Allah.”

Healing the Personal and Communal Wounds of Trauma was another notable theme. One male participant observed that he “gain[ed] uncountable [innumerable] things for example, patience and forgiveness,” while another noted, “the most thing that I got was sharing my problems with my consultant (lay leader). These made me happy.” Participants also expressed general appreciation for how Islamic Trauma Healing reduced their distress, as one member commented “I think this program is the best to heal the hurt people.”

The third central theme was Increasing Accessibility to Help the Community; participants expressed strong desires to grow the program and further disseminate it to the broader Somali community. One participant observed, “It needs more awareness raising and establishing centers for mobilizations.” Another noted, “It is good work because most of our community [has] trauma and we can help them from this program.” In general, the qualitative feedback reinforced the match between Islamic Trauma Healing and the participants' faith, the perceived helpfulness of the program, and the desire to grow the program to help others.

## Discussion

The need for community based, culturally resonant, easily-upscalable, and sustainable models of mental health delivery is high, especially in war-torn, low resource regions such as Somalia. Innovative new models are needed as the reach of our current evidence-based PTSD treatment psychotherapies simply will not permeate areas where they are most needed in the war-torn regions of the Muslim world. Mental health interventions must effectively engage individually and collectively culturally relevant meaning-making mechanisms e.g., (29). For the growing number of refugee and displaced Muslims, the integration of evidence-based practices with Islamic principles is likely necessary to provide a meaningful path to posttraumatic healing. Results of this open trial in Somalia show the strong feasibility and preliminary effectiveness of Islamic Trauma Healing. The lay-led, mosque-based model underlying the intervention truly integrates Islam and evidence-based psychotherapy which is unique in the mental health field. Further, to our knowledge, this is the first clinical trial of any mental health intervention delivered in Somalia. After 6 weeks of group sessions, participants with a range of traumas and PTSD symptoms showed clinically meaningful improvement across well-validated psychiatric and functional indices, with effect sizes similar to benchmarks from expert-delivered Western, individual PTSD psychotherapy (8). Effects were not limited to PTSD symptoms but also showed clinically meaningful reductions in depression and somatic symptoms and improved well-being. Lay leaders, with a brief 2-day training and no experts on the ground, implemented the program successfully in mosques with no drop out or adverse events. Qualitative data from group members highlighted that the program was well-aligned with their faith and captured the need to expand the program to other mosques. This community-based, lay-lead program delivered in the mosques maximizes pre-existing infrastructure, capitalizes on the power of one's faith, and circumnavigates the stigma commonly associated with seeking mental health services.

Any mental health intervention that is going to have reach, particularly where there is little or no infrastructure, must be easily scalable. Islamic Trauma Healing content was carefully vetted to focus on central tenants of Islam, reducing any future sectarian issues and allowing for easy adaptation for other Muslim groups. By task shifting, where existing group leaders train and supervise new group leaders, expert involvement is reduced, costs are lowered, local knowledge is increased, and the program can become self-sustaining (30). Leaders in this trial had no prior mental health experience and training was purposely brief; 2 days with about 6–8 h of direct instruction and role plays. Training was focused on basic discussion group leading skills, making the training itself easily transferrable for lay leaders to train the next generation of group leaders and allowing for exponential program growth to rapidly increase capacity. Fidelity was easily monitored over weekly WhatsApp chats. Programs such as these have the potential to quickly reach hundred and thousands of traumatized individuals. Ultimately, programs such as this answer the clarion call for radical adaptation to meet the local health care resource constraints (10). Somalia is a perfect example of where the mental health ravages of war and natural disaster are great, but the resources and abilities on the ground of outsiders are limited (12).

Any intervention targeting the mental wounds of war also needs to address reconciliation and social community rebuilding, as evidenced by clan violence in Somalia (16). Lack of social support is one of the most powerful factors predicting chronic mental health problems following trauma (31). Protracted civil war and sociopolitical conflict contribute to long-term fragmentation of communities and societies at large, as evidenced by clan-based discord and violence in Somalia. Although societal reconciliation programs show promise in facilitating forgiveness for the perpetration of war atrocities, they may unintentionally exacerbate psychiatric problems (32). Alternatively, mosque-based groups harness the centrality of Islam in peoples' lives and the power of shared religious beliefs and practices. Mosques are often community hubs; placing the program in them builds on this natural capacity. Shared discussion of the effects of trauma and discussion of forgiveness and reconciliation with one another further builds community. Framing this discussion around the lives of the Prophets provides timeless wisdom and a faith-based structure for moving forward after trauma. Finally, community and mosque leadership, not outside experts, promotes community ownership in their own healing and builds community capacity and enthusiasm for larger program reach. Satisfaction and qualitative data from this trial point to these processes emerging. Programs, such as Islamic Trauma Healing, which attend to the social fabric of war-torn, low resource countries and utilize the power of shared beliefs and customs, may be most effective in producing healing and connectedness at both the individual and community level.

This small open trial of Islamic Trauma Healing in Somalia provides additional preliminary evidence, with effect sizes consistent with a previous U.S.-based refugee pilot trial (17), for a radical new model of treating pervasive trauma-related mental health issues in Muslim communities, particularly those in and from war-torn areas or those with few mental health resources (11). A randomized control trial in the U.S. is ongoing to further evaluate efficacy. Lack of a control group, isolating intervention specific effects, and long-term follow-up, showing maintenance of gains, are key limitations. Fidelity of intervention implementation was monitored remotely. Future research will also need to explore adaptation to other Muslim populations and other faiths. The program was explicitly developed to integrate state-of-art science and spiritual practices to address the mental wounds of war, to promote healing, and facilitate reconciliation and connectedness in communities. The community-based model is entirely consistent with the growing recognition of the pervasive impact of trauma as outlined by the UN's sustainable development goals (33) and the WHO's building of mental health and psychosocial support capacity (34). Harnessing the collective powers of the Islamic faith, evidence-based psychotherapeutic science, and social community has the potential to yield an easily scalable, low-cost, self-sustaining, and effective mental health psychosocial intervention for trauma-exposed peoples, ultimately empowering communities to help heal themselves.

## Data Availability Statement

The raw data supporting the conclusions of this article will be made available by the authors, without undue reservation.

## Ethics Statement

The studies involving human participants were reviewed and approved by University of Washington. The patients/participants provided their written informed consent to participate in this study.

## Author Contributions

LZ, JB, NF, DA, and ME designed the study. DA and ME were responsible for study logistics in Somaliland, Somalia, including overseeing coordination of lay leaders, and group implementation (ME). LZ, JB, NF, ME, MD, and AK were responsible for training and supervision. ME, MD, AK, and LZ were responsible for data collection and management. LZ, MD, and AK analyzed the data. LZ, JB, NF, AK, and MD contributed to writing the manuscript. All authors contributed to the article and approved the submitted version.

## Conflict of Interest

The authors declare that the research was conducted in the absence of any commercial or financial relationships that could be construed as a potential conflict of interest.

## References

[1] Kolappa K Henderson DC Kishore SP. No physical health without mental health: lessons unlearned? Bull Health World Organ. (2013) 91:3–3A. 10.2471/BLT.12.115063 23397342 PMC3537253

[2] Cole E Rothblum ED Espin OM. Refugee Women and Their Mental Health: Shattered Societies, Shattered Lives. New York, NY: Routledge (2013). 10.4324/9780203729267

[3] World Health Organization (WHO). A Situation Analysis of Mental Health in Somalia. WHO. (2010). Available online at: http://applications.emro.who.int/dsaf/EMROPUB_2010_EN_736.pdf (accessed December 18, 2019).

[4] United Nations High Commissioner for Refugees (UNHCR). Figures at a Glance. UNHCR. (2019). Available online at: http://www.unhcr.org/en-us/figures-at-a-glance.html (accessed December 18, 2019).

[5] Koenen KC Ratanatharathorn A NG L McLaughlin KA Bromet EJ Stein DJ. Posttraumatic stress disorder in the world mental health surveys. Psychol Med. (2017) 47:2260–74. 10.1017/S0033291717000708 28385165 PMC6034513

[6] Charlson F van Ommeren M Flaxman A Cornett J Whiteford H Saxena S. New WHO prevalence estimates of mental disorders in conflict settings: a systematic review and meta-analysis. Lancet. (2019) 394:240–48. 10.1016/S0140-6736(19)30934-1 31200992 PMC6657025

[7] Song SJ Kaplan C Tol WA Subica A de Jong J. Psychological distress in torture survivors: pre-and post-migration risk factors in a US sample. Soc Psychiatry Psychiatr Epidemiol. (2015) 50:549–60. 10.1007/s00127-014-0982-1 25403567

[8] Cusack K Jonas DE Forneris CA Wines C Sonis J Middleton JC. Psychological treatments for adults with posttraumatic stress disorder: a systematic review and meta-analysis. Clin Psychol Rev. (2016) 43:128–41. 10.1016/j.cpr.2015.10.003 26574151

[9] Neuner F Onyut PL Ertl V Odenwald M Schauer E Elbert T. Treatment of posttraumatic stress disorder by trained lay counselors in an African refugee settlement: a randomized controlled trial. J Consult Clin Psychol. (2008) 76:686–94. 10.1037/0022-006X.76.4.686 18665696

[10] Becker AE Kleinman A. Mental health and the global agenda. N Engl J Med. (2013) 369:66–73. 10.1056/NEJMra1110827 23822778

[11] Bolton P. Global mental health and psychotherapy: importance of task-shifting and a systematic approach to adaptation. In: Stein DJ Bass JK Hofmann SG editors. Global Mental Health and Psychotherapy: Adapting Psychotherapy for Low- and Middle-Income Countries. Cambridge, MA: Academic Press (2019). p. 11–24. 10.1016/B978-0-12-814932-4.00001-X

[12] Pew Forum on Religion & Public Life. U.S. Religious Landscape Survey. Pew Research Center. (2009). Available online at: http://www.pewforum.org/religious-landscape-study/ (accessed December 18, 2019).

[13] Boynton L Bentley J Jackson JC Gibbs TA. The role of stigma and state in the mental health of Somalis. J Psychiatr Pract. (2010) 16:265–68. 10.1097/01.pra.0000386914.85182.78 20644363

[14] Ai AL Tice TN Huang B Ishisaka A. Wartime faith-based reactions among traumatized Kosovar and Bosnian refugees in the United States. Ment Health Relig Cult. (2005) 8:291–308. 10.1080/13674670412331304357

[15] Bentley JA Feeny NC Klein A Dolezal M Zoellner LA. Trauma healing: integrating faith and empirically-supported principles in a community-based program. Cogn Behav Pract. (2021) 28:167–92. 10.1016/j.cbpra.2020.10.005 34025104 PMC8136181

[16] Sakaluk JK Williams A Kilshaw R Rhyner KT. Evaluating the evidential value of empirically supported psychological treatments (ESTs): a meta-scientific review. J Abnorm Psych. (2019) 128:500–9. 10.1037/abn0000421 31368729

[17] Zoellner L Graham B Marks E Feeny N Bentley J Franklin A. Islamic trauma healing: initial feasibility and pilot data. Societies. (2018) 8:47. 10.3390/soc8030047

[18] Lang D Zoellner L Graham B Marks EH Feeny NC. Islahul Qulub: Islamic Trauma Healing. Seattle, WA: University of Washington Center for Anxiety & Traumatic Stress (2016).

[19] Foa E Hembree E Rothbaum BO. Prolonged Exposure Therapy for PTSD: Emotional Processing of Traumatic Experiences, Therapist Guide. New York, NY: Oxford University Press (2007). 10.1093/med:psych/9780195308501.001.0001

[20] Chambers DA Glasgow RE Stange KC. The dynamic sustainability framework: addressing the paradox of sustainment amid ongoing change. Implement Sci. (2013) 8:117. 10.1186/1748-5908-8-117 24088228 PMC3852739

[21] Iwelunmor J Blackstone S Veira D Nwaozuru U Airhihenbuwa C Munodawafa D. Toward the sustainability of health interventions implemented in sub-Saharan Africa: a systematic review and conceptual framework. Implement Sci. (2016) 11:43. 10.1186/s13012-016-0415-5 27005280 PMC4804528

[22] American Psychiatric Association. Diagnostic and Statistical Manual of Mental Disorders, 5th edition, DSM-5. Washington, DC: American Psychiatric Association (2013). 10.1176/appi.books.9780890425596

[23] Zoellner LA Telch M Foa EB Farach FJ McLean CP Gallop R. Enhancing extinction learning in posttraumatic stress disorder with brief daily imaginal exposure and methylene blue: a randomized controlled trial. J Clin Psychiatry. (2017) 78:782–89. 10.4088/JCP.16m10936 28686823

[24] Foa EB McLean CP Zang Y Zhong J Powers MB Kauffman BY. Psychometric properties of the Posttraumatic Diagnostic Scale for DSM-5 (PDS−5). Psychol Assess. (2016) 28:1166–71. 10.1037/pas0000258 26691504

[25] Kroenke K Spitzer RL Williams JB. The PHQ-9. J Gen Intern Med. (2001) 16:606–13. 10.1046/j.1525-1497.2001.016009606.x 11556941 PMC1495268

[26] Gierk B Kohlmann S Kroenke K Spangenberg L Zenger M Brähler E. The somatic symptom scale−8 (SSS-8): a brief measure of somatic symptom burden. JAMA Intern Med. (2014) 174:399–407. 10.1001/jamainternmed.2013.12179 24276929

[27] Bech P Gudex C Johansen KS. The WHO (ten) well-being index: validation in diabetes. Psychother Psychosom. (1996) 65:183–90. 10.1159/000289073 8843498

[28] Larsen DL Attkisson CC Hargreaves WA Nguyen TD. Assessment of client/patient satisfaction: development of a general scale. Eval Program Plann. (1979) 2:197–207. 10.1016/0149-7189(79)90094-6 10245370

[29] Kleinman A. The Illness Narratives: Suffering, Healing and the Human Condition. New York, NY: Basic Books (1988).

[30] World Health Organization (WHO). First Global Conference on Task Shifting. WHO. (2008). Available online at: http://www.who.int/healthsystems/task_shifting/en/ (accessed December 18, 2019).

[31] Brewin CR Andrews B Valentine JD. Meta-analysis of risk factors for posttraumatic stress disorder in trauma-exposed adults. J Consult Clin Psych. (2000) 68:748–66. 10.1037/0022-006X.68.5.748 11068961

[32] Cilliers J Dube O Siddiqi B. Reconciling after civil conflict increases social capital but decreases individual well-being. Science. (2016) 352:787–94. 10.1126/science.aad9682 27174981

[33] World Health Organization (WHO). Mental Health Included in the UN Sustainable Development Goals. WHO. (2015). Available online at: https://www.who.int/mental_health/SDGs/en/ (accessed December 18, 2019).

[34] World Health Organization (WHO). Mental Health Action Plan 2013–2020. WHO. (2013). Available online at: https://www.who.int/mental_health/publications/action_plan/en/ (accessed December 18, 2019).

