# Supplementary material for: Reaching the Unreached: Bridging Islam and Science to Treat the Mental Wounds of War
Source: Front Psychiatry. 2021 Jun 2;12:599293. doi: 10.3389/fpsyt.2021.599293 (PMC8208506; doi:10.3389/fpsyt.2021.599293)
Supplement: Supplementary file 1 [file Data_Sheet_1.docx]

Supplementary Material

# Supplementary Results

**3.4. *Post hoc* Analyses**

Exploratory analyses were conducted examining the potential moderating effects of age and gender for the primary outcome measure of PTSD severity (PDS-5).

On average, men were older (*M* = 31.08, *SD* = 8.81) than women (*M* = 24.64, *SD* = 4.20), *t*(24) 2.44, *p* = .02, *Hedges’ g* = 0.96. However, age was not strongly related to PTSD severity at baseline (*r* = -.28, *p* = .16) or post intervention (*r* = -.06, *p* = .79).

At baseline, women had higher PTSD severity (*M* = 40.00, *SD* = 8.70) than men (*M* = 20.25 *SD* = 10.55), *t*(24) -5.23, *p* < .001, *Hedges’ g* = 2.51. When controlling for baseline PTSD severity, there was no effect of gender on post-intervention PTSD severity, *F*(1, 23) = 0.68, *p* = .42.

Accordingly, there was no strong post hoc evidence that age or gender moderated the effects of the intervention. Small sample size, under powering these analyses, limit their generalizability.
