# Supplementary material for: Reaching the Unreached: Bridging Islam and Science to Treat the Mental Wounds of War
Source: Front Psychiatry. 2021 Jun 2;12:599293. doi: 10.3389/fpsyt.2021.599293 (PMC8208506; doi:10.3389/fpsyt.2021.599293)
Supplement: Supplementary file 2 [file Data_Sheet_2.docx]

**In adeega la Gaadhsiiyo Meelaha Aan la Gaadhin:**

**In Diinta Islaamka iyo Cilimiga Sayniska loo Adeegsado in lagu Daaweeyo Dhaawacyada Maanka ee Dagaalada laga Dhaxlay**

Lori A Zoellner PhD^1^, Jacob A Bentley PhD^2^, Norah C Feeny PhD^3^, Alexandra B Klein MA^3^, Michael L Dolezal MA^2^, Dega A Angula MSc^1^, Momin H Egeh^4,5^

Translated by Dr. Essa Nur Liban^6^

^1^Department of Psychology, University of Washington, Seattle, WA, USA

^2^Department of Clinical Psychology, Seattle Pacific University, Seattle, WA, USA

^3^Department of Psychological Sciences, Case Western Reserve University, Cleveland, OH, USA

^4^Ma’alin Haruon Masjid, Hargeisa, Somaliland

^5^Abu-Bakar Al-Siddique Islamic Center, Borama, Somaliland

^6^Vice Chancellor, University of Burao, Somaliland

**Soo Koobida Xogta Daraasadan**

Waxa loo baahanyahay in habab cusub lagu xoojiyo dadka aan xirfadaha gaarka lahayn taas oo bulshada wax loogu qabanaayo qaab ku salaysan diintooda si loo wajaho qof ama bulshada inta la kulantay dhawacyo/xanuuno xagga maanka oo ay ka dhaxleen dagaalada ama iska hormiaadada bulshada dhexdeeda ka dhacay. Dalalka ay ka dhaceen dagaalo sokeeye sida Soomaaliya waxa jooga dad fara badan oo leh xanuunada laga dhaxlo dagaalada oo raad ku leh maanka binaadamka, hasa yeeshee waxa ka qabashada xanuunadaas waxaa hortaagan ama carqald ku ah a) iyada oo la lala’yahay dadkii bukaanka caawin lahaa, b) iyada oo cuqdada laga qaboo dadkaas, c) iyo iyada oo aan lala xidhiidhin xanuunka qofkaa dhinca diintiisa uu rumaysanyahay. Haddaba qaabkii hore iyo kan dambe ee la isku deyay in arrimahaa lagu daaweeyo (NCT03761732) wuxuu ku salysnaa in Imaamada Masajidayadu ku hawlgalaan qaabka loo bixiyay “*ku bugsii dadka isku buuqsan qaabka islaamiya” (Islamic Trauma Healing)* iyaga oo bartilmaayaysanaaya dadka maskaxiyan waxyeelo ka soo gaadhay dagaaladii iyo sidii uu dib mujtamaca uga mid noqon lahaa (*N* = 26) ee dalka Soomaaliland iyo Soomaaliya. Imaamada hawlwadeenda ahi waxay qaateen laba (2) casho oo tababar ah iyada oo lagala socdo dhinaca mareegta WhatsAppka. Lixadaa kulan ee waxbarsho waxay si dhabaha u taabanaysaa in si dhabah loo daaweeyo dadkaa iyada oo xooga la saaraayo caafimaadinta maanka iyo mabaadiidada ku qotonta islaamka iyada oo la adeegsanaayo xikmadihii qaab nololeedkii nebiyada iyo in Illaahay loo laabto oo lagu ducaysto in illaahay bogsiiyo. Waxa jiray natiijo balaaadhan oo laga arkay daawaynta (PTSD [g = 1.91], niyadjabka [g = 2.00], boonbuunita dareenka xanuunada jidhka [g = 2.73], iyo badhaadhaha nafta [g = 1.77]). Markii la darsay xogtii laga soo uruiyay kooxaha waxay iftiiniyeen sida wanaagsan ee barnaamijkan u waafaqsanyayahy waxay rumaysanaayeen, dhisida mujtamaca iyo in loo baahanyayahy in la sii balaadhiyo. Natiijadan darsaaasdani waxay muujisay suurtagalinimada in barnaamijakani yahay mid loo isticimaali karo wadamada ay dagaalaadu burburiyeen ee islaamka ah iyo mujtamacyada qaxootiyada ah, barnaamajikan oo ah mid aan u baahnanyn khubaro u gaara islamarkaana ah mid si dhib yar loo horumarin karo. Haddaba barnaamijan waa mid mustaqbal fiican oo kharash fara badana aan ku kacayn, waxaan la filayaa inuu mustaqbalka iskii u sii socon karo isga oo ka dhimbiil qaadanaaya habraacaa qaabka isku daawaynta islaamka, isla marakaana wajahaaya inuu daaweeyo dadka dagaaladu maankooda waxyeeleeyeen iyada oo waafaqasan baaqii IOM ka ee ku aadanaa in horuumar lagu sameeyo sidii wax looga qaban lahaa baahiyaha daawayneed ee aan weli la taaban.
